# Effects of Hyperbaric Oxygen at 1.25 Atmospheres Absolute with Normal Air on Macrophage Number and Infiltration during Rat Skeletal Muscle Regeneration

**DOI:** 10.1371/journal.pone.0115685

**Published:** 2014-12-22

**Authors:** Naoto Fujita, Miharu Ono, Tomoka Tomioka, Masataka Deie

**Affiliations:** 1 Graduate School of Biomedicine and Health Sciences, Hiroshima University, Hiroshima City, Hiroshima, Japan; 2 Faculty of Medicine, Hiroshima University, Hiroshima City, Hiroshima, Japan; Rutgers University -New Jersey Medical School, United States of America

## Abstract

Use of mild hyperbaric oxygen less than 2 atmospheres absolute (2026.54 hPa) with normal air is emerging as a common complementary treatment for severe muscle injury. Although hyperbaric oxygen at over 2 atmospheres absolute with 100% O_2_ promotes healing of skeletal muscle injury, it is not clear whether mild hyperbaric oxygen is equally effective. The purpose of the present study was to investigate the impact of hyperbaric oxygen at 1.25 atmospheres absolute (1266.59 hPa) with normal air on muscle regeneration. The tibialis anterior muscle of male Wistar rats was injured by injection of bupivacaine hydrochloride, and rats were randomly assigned to a hyperbaric oxygen experimental group or to a non-hyperbaric oxygen control group. Immediately after the injection, rats were exposed to hyperbaric oxygen, and the treatment was continued for 28 days. The cross-sectional area of centrally nucleated muscle fibers was significantly larger in rats exposed to hyperbaric oxygen than in controls 5 and 7 days after injury. The number of CD68- or CD68- and CD206-positive cells was significantly higher in rats exposed to hyperbaric oxygen than in controls 24 h after injury. Additionally, tumor necrosis factor-α and interleukin-10 mRNA expression levels were significantly higher in rats exposed to hyperbaric oxygen than in controls 24 h after injury. The number of Pax7- and MyoD- or MyoD- and myogenin-positive nuclei per mm^2^ and the expression levels of these proteins were significantly higher in rats exposed to hyperbaric oxygen than in controls 5 days after injury. These results suggest that mild hyperbaric oxygen promotes skeletal muscle regeneration in the early phase after injury, possibly due to reduced hypoxic conditions leading to accelerated macrophage infiltration and phenotype transition. In conclusion, mild hyperbaric oxygen less than 2 atmospheres absolute with normal air is an appropriate support therapy for severe muscle injuries.

## Introduction

Muscle regeneration is an adaptive response after injury that follows a well-defined series of events. Although there are numerous causes for muscle injury, such as sport, trauma, and surgery, the events in the regenerative process are common regardless of the causes. During adaptive response to injury, satellite cells, which represent a pool of quiescent muscle precursors, are activated, proliferate, differentiate, and fuse to form new muscle fibers [1], [2]. This series of events is controlled by macrophages that infiltrate the injured area [1], [2], [3], [4]. Macrophages can be classified into two subtypes according to the expression of specific cell markers [2]. M1 macrophages that express CD68 promote the activation and proliferation of satellite cells, and remove necrotic debris [5]. M2 macrophages that express CD68 and CD206 promote the differentiation and fusion of satellite cells [1], [2], [3], [4]. As muscle regeneration progresses from the degenerative to the regenerative stages, the M1 macrophages transition from an M1 pro-inflammatory to an M2 anti-inflammatory phenotype [6], [7]. Previously, we reported that macrophage activity is regulated by therapeutic treatments such as ice and heat, which affect the progression of muscle regeneration [8], [9]. Therefore, macrophage function must be investigated to devise effective treatments that promote muscle regeneration.

Exposure to hyperbaric oxygen is widely accepted as a method to promote healing of bone fracture [10], articular cartilage injury [11], spinal cord injury [12], and skeletal muscle injury [13], [14], [15]. For the treatment of muscle injury, Best *et al*. showed that hyperbaric oxygen at 2.5 atmospheres absolute (ATA; 2533.18 hPa) with 100% O_2_ accelerates morphological regeneration after injury [14]. Similarly, Gregorevic *et al*. reported that hyperbaric oxygen at 3 ATA (3039.82 hPa) with 100% O_2_ increases the contractile property of injured muscle during the regeneration process [15]. Although the effectiveness of hyperbaric oxygen in the treatment of various medical conditions is proven and established, access to facilities that can provide hyperbaric oxygen therapy is severely limited. Mild hyperbaric oxygen with normal air has emerged recently as an accepted complementary treatment for muscle injury in sport medicine. Furthermore, this method is gaining popularity as a home remedy to further improve recovery from muscle injury. However, it remains to be established whether exposure to mild hyperbaric oxygen is equally effective to hyperbaric oxygen treatment at improving muscle healing after traumatic injury. Should mild hyperbaric oxygen promote muscle regeneration, it could provide an additional therapy for muscle injury. The purpose of the present study was to determine whether mild hyperbaric oxygen with normal air is beneficial to recovery from muscle injury, and to investigate the role of macrophages during muscle regeneration.

## Materials and Methods

### Experimental design

This study was approved by the Institutional Animal Care and Use Committee of Hiroshima University (A13–132), and was carried out according to the Hiroshima University Regulations for Animal Experimentation. All experiments were conducted in accordance with the National Institute of Health (NIH) Guidelines for the Care and Use of Laboratory Animals (National Research Council, 1996).

Sixty adult male Wistar rats, weighing 235–256 g, were used in the present study. The rats were anesthetized by intraperitoneal injection of sodium pentobarbital (40 mg/kg body weight). To induce muscle injury, the rats were injected with 0.5 mL of bupivacaine hydrochloride 0.5% in the tibialis anterior muscle. After the intramuscular injection of bupivacaine hydrochloride, all the animals were randomly assigned to hyperbaric oxygen (HB, n = 30) experimental or non-hyperbaric oxygen (Non-HB, n = 30) control groups. Rats in the HB group were exposed to 1.25 ATA (1266.59 hPa), and the treatment was started immediately after the bupivacaine hydrochloride injection and continued uninterrupted for 28 days, with the exception of breaks required for daily cage cleaning. The cages in the Non-HB group were placed in a hyperbaric chamber at normal atmospheric pressure (1013.27 hPa). All animals were housed in a controlled environment with a fixed 12-h light∶dark cycle and at a constant temperature of 22±2°C. Food and water were provided *ad libitum*. The rats were sacrificed with an overdose of sodium pentobarbital at 24 and 48 h, 5, 7, 14, and 28 days after injury (n = 5 in each group at each time point). The injured muscles were immediately removed, frozen in liquid nitrogen-cooled isopentane, and stored at −80°C until the analyses.

### Histological analysis

Transverse sections at a thickness of 10 µm were obtained using a cryostat from the middle part of the tibialis anterior muscle and mounted on amino silane-coated glass slides. The sections were stained with hematoxylin and eosin for histological observation. The cross-sectional areas of muscle fibers with centrally located nuclei, which were considered to be regenerating after injury, were measured using the ImageJ software (NIH, MD, USA). The cross-sectional areas of more than 200 muscle fibers exhibiting centrally located nuclei were measured per animal.

For immunofluorescence, the sections were fixed in acetone at −20°C and blocked with Blocking One Histo (Nakalai Tesque, Kyoto, Japan). The sections were then incubated at 4°C overnight with anti-dystrophin (1∶200, sc-15376; Santa Cruz Biotechnology, TX, USA) and anti-CD68 (1∶200, sc-59103; Santa Cruz Biotechnology) antibodies to identify M1 macrophages, anti-CD 206 (1∶100, sc-48758; Santa Cruz Biotechnology) antibody to identify M2 macrophages, anti-Pax7 (1∶100, sc-81648; Santa Cruz Biotechnology) antibody to identify satellite cells, anti-MyoD (1∶100, sc-760; Santa Cruz Biotechnology) antibody to identify activated satellite cells, and anti-myogenin (1∶100, sc-12732; Santa Cruz Biotechnology) antibody to identify differentiated satellite cells. Subsequently, sections were exposed to Alexa Fluor 488- or 555-conjugated anti-rabbit or anti-mouse immunoglobulin G (1∶2,000; Cell Signaling, MA, USA) for 60 min at room temperature, and mounted with medium containing 4′,6-diamidino-2-phenylindole (DAPI; H-1500, Vector Laboratories). Parallel slides processed without the primary antibody served as negative controls. The sections were analyzed and images were acquired on a fluorescence microscope (BZ-9000, Keyence, Osaka, Japan). The number of CD68+/DAPI+, CD68+/CD206+/DAPI+, Pax7+/MyoD-/DAPI+, Pax7+/MyoD+/DAPI+, Pax7-/MyoD+/DAPI+, MyoD+/myogenin+/DAPI+, and MyoD-/myogenin+/DAPI+ nuclei were quantified for 5 fields per muscle. All measurements were carried out with ImageJ software (NIH).

### Quantitative polymerase chain reaction (qPCR) analysis

Total RNA was isolated from ∼20 mg of each muscle using a lysis buffer containing 0.5 mL of TRIzol reagent (15596-026, Invitrogen, Tokyo, Japan). Reverse transcription was carried out using the High-Capacity cDNA Reverse Transcription Kit (4374966, Applied Biosystems, CA, USA), and the resultant cDNA samples were stored at −20°C.

Expression levels of TNF-α (Rn01525859_g1) and IL-10 (Rn00563409_m1) mRNA were quantified using the TaqMan Gene Expression Assays (Applied Biosystems). Each TaqMan probe and primer set was validated by performing qPCR with a series of cDNA template dilutions to obtain standard curves of threshold cycle time against relative concentration using the normalization gene 18S (Rn03928990_g1). qPCR was performed using the PCR Fast Advanced Master Mix (Applied Biosystems) in a 96-well reaction plate. Each well contained 1 µL of cDNA template, 5 µL of PCR Fast Advanced Master Mix, 3.5 µL of RNase-free water, and 0.5 µL of TaqMan Gene Expression Assays in a reaction volume of 10 µL. All samples and nontemplate control reactions were performed in CFX96 (BioRad, CA, USA) under the following conditions: 50°C for 2 min, 95°C for 20 s, followed by 40 cycles at 95°C for 3 s and 60°C for 30 s.

### Western blot analysis

The frozen muscle samples were homogenized in ice-cold homogenizing buffer containing 50 mM Tris-HCl, pH 7.8, 0.15 M NaCl, and 1% protease inhibitor cocktail (25955-11, Nacalai tesque). The homogenates were centrifuged at 18,000*×g* for 30 min at 4°C. Total protein concentration was determined using a protein determination kit (500-0006, BioRad). The homogenate were solubilized in a sample loading buffer containing 0.5 M Tris-HCl, pH 6.8, 2% sodium dodecyl sulfate (SDS), 10% glycerol, 5% β-mercaptoethanol, and 0.005% bromophenol blue. The samples were boiled for 10 min at 80°C. Forty micro grams of the protein sample was loaded on 10 or 12.5% SDS-polyacrylamide gel, and after 70 min of electrophoresis at 40 mA, the proteins were transferred to a polyvinylidene fluoride membrane for 120 min at 200 mA. The membranes were blocked using a blocking reagent (Blocking one, Nakalai Tesque) for 60 min, and then incubated with anti-MyoD (1∶200, sc-760; Santa Cruz Biotechnology) and anti-myogenin (1∶200, sc-12732; Santa Cruz Biotechnology) antibodies at 4°C overnight. Subsequently, the membranes were incubated for 60 min at room temperature with anti-rabbit (1∶10,000, sc-2370; Santa Cruz Biotechnology) or anti-mouse IgG (1∶10,000, 012-23641; Wako, Osaka, Japan) antibody conjugated to horseradish peroxidase. The signals were detected using a chemiluminescence detector (ECL Prime, GE Healthcare, NJ, USA) and analyzed with an image reader (Versa Doc 5000, BioRad).

### Statistical analysis

Data were expressed as means ± standard error. The significance of differences between the groups was evaluated using the Independent *t*-test, and *p*<0.05 was considered statistically significant.

## Results

### Cross-sectional area of centrally nucleated muscle fibers

**Figure 1:**
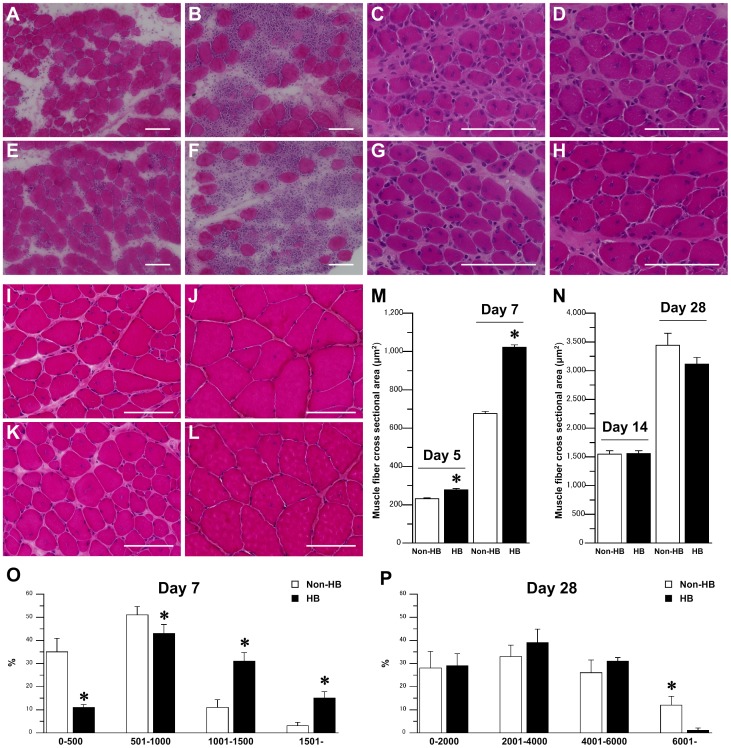
Skeletal muscle regeneration after injury. Representative muscle fiber cross-sections stained with hematoxylin and eosin in the non-hyperbaric (Non-HB; A–D, I, J) and hyperbaric (HB; E–H, K, L) groups at 24 (A, E) and 48 (B, F) h, and 5 (C, G), 7 (D, H), 14 (I, K), and 28 (J, L) days after injury. Bar  = 100 µm. Cross-sectional area of centrally nucleated muscle fibers 5 and 7 days (M), and 14 and 28 days (N) after injury. Distribution of centrally nucleated muscle fiber cross-sectional area at 7 (O) and 28 (P) days after injury. Three sections per animal and 3 randomly chosen fields per section were evaluated. In each section, over 50 centrally nucleated muscle fibers were measured. Values represent means ± standard error (SE). * is significantly different from the Non-HB group, *p*<0.05.

Degeneration of muscle fibers was observed 24 h after injury in both the HB and Non HB groups (Fig. 1A and E). Accordingly, infiltration of leukocytes was reported at 24 h, and achieved its peak 48 h after injury in the both groups (Fig. 1B and F). Centrally nucleated muscle fibers were detectable 5 days after injury (Fig. 1C and G), and the fibers increased in size after 7 days in both the groups (Fig. 1D and H). There were no significant differences in the number of muscle fibers with centrally located nuclei per mm^2^ between the Non-HB and HB groups at 5 (Non-HB: 460±4, HB: 374±4) and 7 (Non-HB: 321±5, HB: 315±4) days after injury. The number of muscle fibers with centrally located nuclei decreased gradually during the late phase after injury in both the groups (Fig. 1I–L). The cross-sectional area of centrally nucleated muscle fibers was significantly larger in the HB group than in the Non-HB group 5 and 7 days after injury (Fig. 1M). However, there were no significant differences between the Non-HB and HB groups at 14 and 28 days after injury (Fig. 1N). Larger centrally nucleated muscle fibers were observed in the HB group 7 days after injury, and the percentage of these muscle fibers was higher in the HB group than in the Non-HB group (Fig. 1O). However, the percentage of these muscle fibers was almost equal between the Non-HB and HB groups at 28 days after injury (Fig. 1P).

### Number of M1 and M2 macrophages and expression levels of TNF-α and IL-10 mRNA

**Figure 2:**
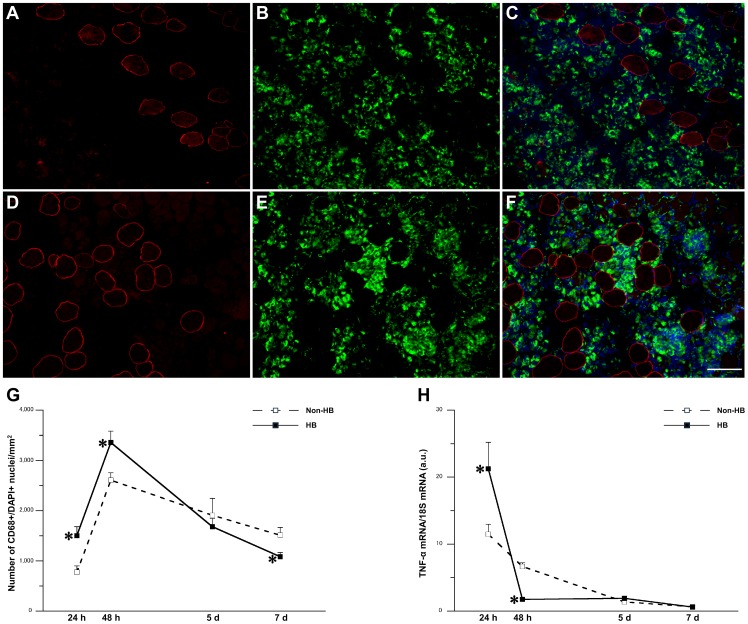
Number of M1 macrophages and expression level of tumor necrosis factor (TNF)-α mRNA. Representative immunofluorescence staining for dystrophin (red; A, D), CD68 (green; B, E), and 4′,6-diamidino-2-phenylindole (DAPI; blue), and merged images of signals from both cell markers (C, F) in the Non-HB (A–C) and HB (D–F) groups 48 h after injury. Bar  = 100 µm. Number of CD68+/DAPI+ nuclei per mm^2^ (G). Three sections per animal and 5 randomly chosen fields per section were evaluated. The expression level of TNF-α mRNA (H). N = 5 in each group at each time point. Values were calculated as fold changes relative to healthy control rats and represent means ± SE. * is significantly different from the Non-HB group, *p*<0.05.

Infiltration of CD68-positive M1 macrophages was detectable 24 h after injury and reached its peak 48 h after injury in both the groups (Fig. 2A–F). The number of CD68-positive cells per mm^2^ was significantly higher in the HB group than in the Non-HB group 24 and 48 h after injury (Fig. 2G). Conversely, there were significantly fewer CD68-positive cells per mm^2^ in the HB group than in the Non-HB group 7 days after injury. The expression level of TNF-α mRNA was significantly higher in the HB group than in the Non-HB group 24 h after injury. Conversely, the expression level of TNF-α mRNA was significantly lower in the HB group than in the Non-HB group 48 h after injury (Fig. 2H).

**Figure 3:**
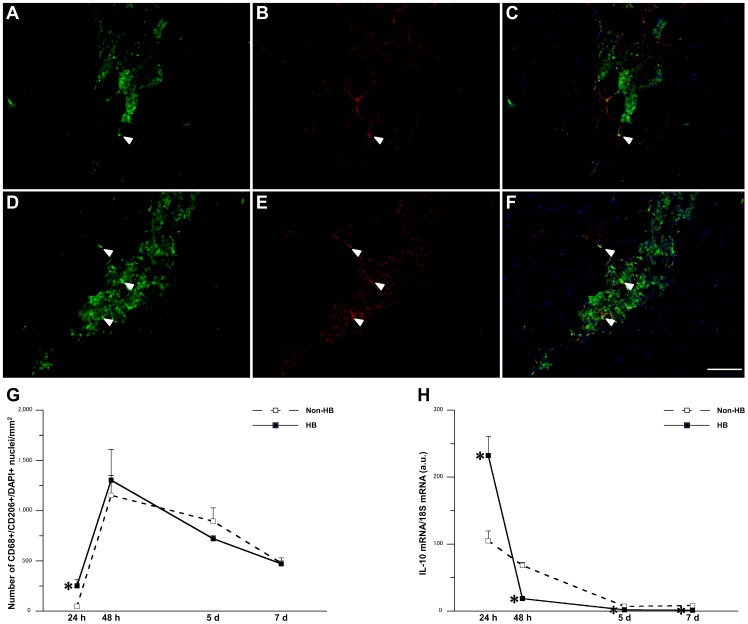
Number of M2 macrophages and expression level of interleukin (IL)-10 mRNA. Representative immunofluorescence staining for CD68 (green; A, D), CD206 (red; B, E), and DAPI (blue), and merged images of signals from both cell markers (C, F) in the Non-HB (A–C) and HB (D–F) groups 24 h after injury. Arrowheads indicate representative CD68- and CD206-positive nuclei. Bar  = 100 µm. Number of CD68+/CD206+/DAPI+ nuclei per mm^2^ (G). Three sections per animal and 5 randomly chosen fields per section were evaluated. The expression level of IL-10 mRNA (H). N = 5 in each group at each time point. Values were calculated as fold changes relative to healthy control rats and represent means ± SE. * is significantly different from the Non-HB group, *p*<0.05.

Although infiltration of CD68- and CD206-positive M2 was observed 24 h after injury in the HB group, the effect was marginal in the Non-HB group (Fig. 3A–F). Accordingly, the HB group exhibited significantly higher numbers of CD68- and CD206-positive cells per mm^2^ than in the Non-HB group 24 h after injury (Fig. 3G). The expression level of IL-10 mRNA was significantly higher in the HB group than in the Non-HB group 24 h after injury. Conversely, the expression level of IL-10 mRNA was significantly lower in the HB group than in the Non-HB group 48 h, and 5 and 7 days after injury (Fig. 3H).

### Number of activated or differentiated satellite cells and expression levels of MyoD and myogenin proteins

**Figure 4:**
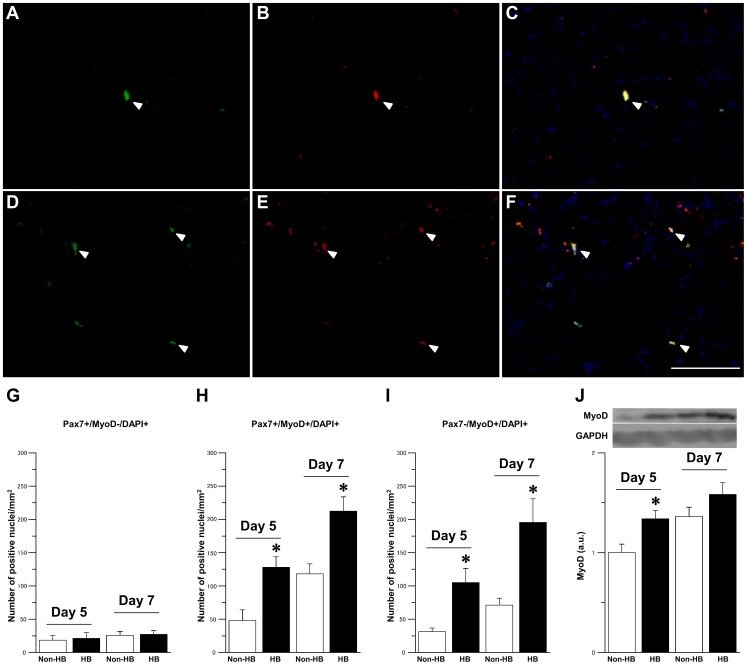
Satellite cells activation. Representative immunofluorescence staining for Pax7 (green; A, D), MyoD (red; B, E), and DAPI (blue), and merged images of signals from both cell markers (C, F) in the Non-HB (A–C) and HB (D–F) groups 7 days after injury. Arrowheads indicate representative Pax7- and MyoD-positive nuclei. Bar  = 100 µm. Number of Pax7+/MyoD-/DAPI+ (G), Pax7+/MyoD+/DAPI+ (H), and Pax7-/MyoD+/DAPI+ (I) nuclei per mm^2^. Three sections per animal and 5 randomly chosen fields per section were evaluated. The expression level of MyoD protein and representative western blot (J). N = 5 in each group at each time point. Values were calculated as fold changes relative to the Non-HB group at 5 day after injury and represent means ± SE. * is significantly different from the Non-HB group, *p*<0.05.

Pax7-and MyoD-positive satellite cells, representing the activated and proliferating cell population, were observed 5 days after injury, and their numbers increased after 7 days in both the groups (Fig. 4A–F). There was no significant difference in the number of Pax7+/MyoD- nuclei per mm^2^ between the Non-HB and HB groups at 5 and 7 days after injury (Fig. 4G). However, the number of Pax7+/MyoD+ and Pax7-/MyoD+ nuclei per mm^2^ was significantly higher in the HB group than in the Non-HB group 5 and 7 days after injury (Fig. 4H and I). The expression level of MyoD protein was significantly higher in the HB group than in the Non-HB group 5 days after injury (Fig. 4J).

**Figure 5:**
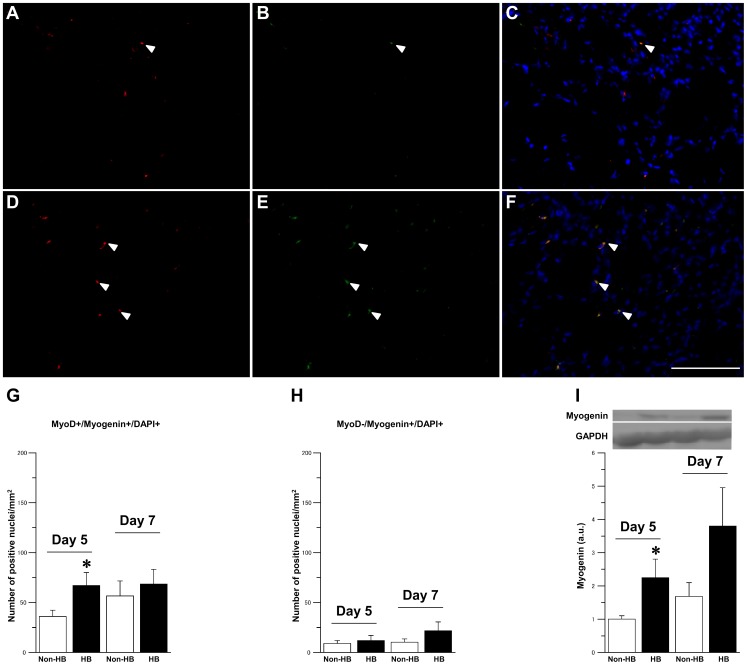
Satellite cells differentiation. Representative immunofluorescence staining for MyoD (red; A, D), myogenin (green; B, E), and DAPI (blue), and merged images of signals from both cell markers (C, F) in the Non-HB (A–C) and HB (D–F) groups 5 days after injury. Arrowheads indicate representative MyoD- and myogenin-positive nuclei. Bar  = 100 µm. Number of MyoD+/myogenin+/DAPI+ (G) and MyoD-/myogenin+/DAPI+ (H) nuclei per mm^2^. Three sections per animal and 5 randomly chosen fields per section were evaluated. The expression level of myogenin protein and representative western blot (I). N = 5 in each group at each time point. Values were calculated as fold changes relative to the Non-HB group at 5 day after injury and represent means ± SE. * is significantly different from the Non-HB group, *p*<0.05.

Differentiated MyoD- and myogenin-positive satellite cells were detected 5 days after injury in both the groups (Fig. 5A–F). The number of MyoD+/myogenin+ nuclei per mm^2^ was significantly higher in the HB group than in the Non-HB group at 5 days and remained stable 7 days after injury in both the groups (Fig. 5G). There was no significant difference in the number of MyoD-/myogenin+ nuclei per mm^2^ between the Non-HB and HB groups at 5 and 7 days after injury (Fig. 5H). The expression level of myogenin protein was significantly higher in the HB group than in the Non-HB group at 5 day after injury (Fig. 5I).

## Discussion

The results of the present study suggest that hyperbaric oxygen at 1.25 ATA with normal air enhances muscle regeneration after injury. The first phase of muscle regeneration, also known as the proliferative stage, entails the transient expression of the muscle-specific transcription factor MyoD by quiescent satellite cells, which leads to cell activation and proliferation [16], [17]. In the second phase, otherwise known as the differentiation stage, the muscle-specific transcription factor myogenin is co-expressed with MyoD until the satellite cells fuse with each other to differentiate into the multinucleated muscle cells that characterize mature muscle fibers [16]. Expression levels of MyoD and myogenin were higher in the context of exposure to hyperbaric oxygen than under normoxic conditions, suggesting that hyperbaric oxygen accelerates healing of injured muscles. Interaction of M1 with M2 macrophages is critical for the regeneration process, and secretion of cytokines such as TNF-α and IL-6 from M1 macrophages is necessary for progression from the proliferative to the differentiation stage [2]. We report that hyperbaric oxygen accelerated infiltration of M1 macrophages and satellite cells activation, events that possibly promoted the progression of the regeneration process to the proliferative stage. In addition to M1 macrophage infiltration, transition from M1 to M2 macrophages is observed during the progression from the proliferative to the differentiation stage [18], indicating that the transition of the macrophage phenotype is critical for muscle regeneration. M2 macrophages secrete anti-inflammatory cytokines such as IL-10 and allow progression of the differentiation stage [2], [6]. We demonstrated that hyperbaric oxygen accelerated M2 macrophage infiltration at the early phase after injury, suggesting that this treatment promotes the transition from the M1 to M2 macrophage phenotypes and, as a consequence, progression to the differentiation stage.

Conditions in the microenvironment of necrotic tissue are characterized by hypoxia [19], [20], and the efficacy of hyperbaric oxygen on muscle regeneration is mediated by increased dissolved oxygen levels at the site of muscle degeneration. Hypoxia inhibits the expression of chemokines such as monocyte chemotactic protein-1 and attenuates macrophage chemotaxis [20], [21]. Additionally, although the expression levels of MyoD and myogenin mRNA increased in the muscles after injury, this activation was impaired under hypoxic conditions [22]. The hyperbaric oxygen treatment carried out in the present study possibly increased the dissolved oxygen levels in blood, an event that might extend the oxygen diffusion distance and elevate the oxygen diffusion rate at the site of muscle injury. Previous studies have reported that exposure to hyperbaric oxygen increases the dissolved oxygen concentration in the arterial blood [23] and elevates cellular oxygen level [24]. We observed that opposing hypoxia by treatment with hyperbaric oxygen accelerated the infiltration of macrophages, possibly resulting in accelerated proliferation of satellite cell, transition of the macrophage phenotype, and differentiation of satellite cells. However, we did not measure the oxygen level in the muscle tissue in this study. Therefore, the influence of oxygenation on muscle injury repair would need to be analyzed further.

Although we reported that hyperbaric oxygen is beneficial to muscle regeneration, the efficacy of this therapy is controversial [25], [26], [27], [28]. A negative effect of hyperbaric oxygen on muscle regeneration was observed in the context of muscle injury induced by eccentric contractions, also known as delayed onset muscle soreness. However, strong eccentric contractions cause muscle injury without leading to a severe inflammatory response or macrophage infiltration, so that muscle damage occurs only at the ultrastructural level and affects only the myofibrils and the Z line [29], [30], [31]. We speculate that hyperbaric oxygen might be an effective therapy only when muscle injury is associated with severe inflammation, due to the acceleration of macrophage infiltration caused by increased oxygen levels in the damaged tissue. In contrast to our findings, other laboratories have reported that hyperbaric oxygen has no effect on the expression of MyoD and myogenin after muscle injury induced by bupivacaine hydrochloride [32]. However, treatment with hyperbaric oxygen was carried out only once a day for 1 h, and this regimen was probably insufficient to observe the beneficial effects of hyperbaric oxygen on muscle regeneration. Macrophages infiltrate the site of muscle damage following neutrophil invasion, and peak number of macrophages is not observed until 48 h post-injury [2]. We propose that hyperbaric oxygen as a strategy to promote healing of severe muscle injury associated with macrophage infiltration should be initiated as soon as possible after the onset of muscle injury, and should be conducted for at least 48 h to ensure efficacy.

In conclusion, mild hyperbaric oxygen attenuates hypoxia, thereby promoting skeletal muscle regeneration after injury, due to acceleration of macrophage infiltration and phenotype transition. Gregorevic *et al*. reported that hyperbaric oxygen enhances muscle regeneration, and that the efficacy of this treatment is greater at 3 ATA than at 2 ATA [33]. We demonstrated that hyperbaric oxygen promotes muscle regeneration even at 1.25 ATA with normal air, suggesting that mild hyperbaric oxygen is a viable adjuvant therapy to promote healing of severe muscle injury. This study demonstrated the efficacy of hyperbaric oxygen on muscle regeneration. However, the study has some limitations. M2 macrophages are typically absent or present at low levels at 1–2 days after injury, and their numbers attain a peak value at 4–5 days after injury. However, the number of CD68– and CD206-positive cells reached its peak 48 h after injury and the kinetics overlapped that of CD68-positive cells in the present study. Although the reason for this remains unclear, CD68-positive cells might include M1 macrophages but both M1 and M2 macrophages. In the future study, we need to perform co-staining with M1 specific marker excluding CD68 and CD206. Additionally, the experimental model might be causally related to the problem. We used an experimental muscle injury model by bupivacaine hydrochloride injection in the present study. This method induces extremely severe injury and is not clinically representative. Additionally, the efficacy of hyperbaric oxygen on muscle regeneration was limited to the early phase after injury, and the size of the regenerating muscle fiber at the late phase after injury was almost the same between the groups. Therefore, further research is required to determine the effect of hyperbaric oxygen on clinically relevant muscle injury.
